# Editorial: Revealing the unconventional mechanisms of mitochondria-targeting drugs in heart-related diseases

**DOI:** 10.3389/fphar.2024.1543291

**Published:** 2025-01-06

**Authors:** Kaidi Ren, Yang Yang

**Affiliations:** ^1^ Department of Pharmacy, The First Affiliated Hospital of Zhengzhou University, Zhengzhou, China; ^2^ Clinical Systems Biology Laboratories, The First Affiliated Hospital of Zhengzhou University, Zhengzhou, China

**Keywords:** mitochondria, drug, heart, heart disease, mitochondria al pathway

The heart, as the body’s largest energy consumer, is highly dependent on mitochondrial function to sustain ATP production (Da Dalt et al., 2023). Mitochondrial dysfunction is a central factor in the development and progression of cardiovascular diseases. Traditional treatment strategies have largely focused on addressing oxidative stress, enhancing mitochondrial biosynthesis, and correcting mitochondrial iron homeostasis (Liu et al., 2022). However, emerging research on mitochondria-targeted therapies is uncovering innovative mechanisms that go beyond these conventional approaches. For example, modulating mitochondrial dynamics, such as regulating mitophagy or stabilizing the mitochondrial protein balance, has shown promising potential in protecting heart cells from damage and facilitating recovery (Ajoolabady et al., 2022). Additionally, the role of mitochondria in cellular signaling pathways and apoptosis is gaining attention, with some therapies targeting mitochondria-mediated cell death pathways to mitigate ischemic damage or slow the progression of heart failure (He et al., 2020). These novel therapeutic strategies, which focus on modifying the behavior of mitochondria at the cellular level, present more targeted and effective methods for addressing the root causes of heart diseases rather than just alleviating symptoms. Understanding the complex interactions among mitochondrial energy metabolism, iron handling, and cellular signaling networks is crucial for developing the next-generation of treatments that could fundamentally transform cardiovascular disease management.

This Research Topic brings together several significant studies that explore the pivotal role of mitochondria in the pathogenesis of heart diseases and the potential of mitochondria-targeting therapies. The heart, which is the largest energy consumer in the human body, heavily depends on mitochondrial function. Dysfunction in mitochondria plays a central role in the progression of heart diseases, making it a critical area of research for therapeutic development. Kun et al. reviewed the use of ketotherapy in heart failure, highlighting how ketone metabolism improves mitochondrial function, reduces oxidative stress, and offers therapeutic potential for heart failure. Min et al. further investigated therapeutic strategies, focusing on the protective effects of Sterofundin, a widely used electrolyte solution, on myocardial infarction. These findings demonstrate that Sterofundin reduces oxidative stress and apoptosis by activating autophagy, ultimately protecting the heart from post-MI damage. In addition, Pengjie et al. applied advanced fetal heart quantification (Fetal HQ) to examine the impact of gestational diabetes mellitus (GDM) on fetal heart function and reported that GDM is associated with impaired right ventricular function, particularly in the later stages of pregnancy, which opens new possibilities for early heart disease prediction. Yuhu et al. took a deeper look at mitochondrial quality control by exploring the role of FUNDC1-mediated mitophagy in cardioprotection, explaining how it prevents the accumulation of damaged mitochondria and excessive mitophagy, which could lead to cell death. Finally, Shu-Ang et al. reviewed the application of pH-sensitive fluorescent proteins in cellular imaging, especially for monitoring mitochondrial pH changes, which can reveal the relationship between mitochondrial dysfunction and heart disease progression.

These studies collectively increase our understanding of the intricate ways in which mitochondrial dysfunction drives the progression of heart diseases. These findings highlight the potential of mitochondrion-targeted therapies, ranging from metabolic regulation and autophagy modulation to cutting-edge imaging techniques, to reshape the future of heart disease diagnostics and treatment strategies. By investigating diverse therapeutic approaches, such as ketone-based therapies, autophagy activation, and mitophagy regulation, these studies propose new therapeutic avenues that could significantly improve clinical outcomes. Furthermore, the integration of advanced imaging technologies, such as pH-sensitive fluorescent proteins, opens up exciting possibilities for real-time monitoring of mitochondrial function, providing deeper insights into disease mechanisms and treatment efficacy. Together, these articles not only refine our knowledge of the role of mitochondria in cardiovascular diseases but also set the stage for novel therapeutic innovations that could have a lasting impact on heart disease management and prevention.

As research has revealed the diverse roles of mitochondrial dysfunction in heart diseases such as heart failure, myocardial infarction, and ischemic conditions, the potential of mitochondria-targeting therapies to revolutionize clinical practice has become increasingly clear. These therapies, including metabolic modulation, autophagy enhancement, and advanced imaging techniques, could offer significant advantages by addressing the underlying causes of disease rather than just alleviating symptoms. For example, ketone-based therapies have shown promise in heart failure treatment by improving mitochondrial function, reducing oxidative stress, and enhancing cellular energy production, which may stabilize cardiac function and reduce the burden on a failing heart (Takahara et al., 2022). Similarly, autophagy activation, as demonstrated in studies on Sterofundin, could mitigate myocardial infarction damage by promoting mitochondrial turnover and reducing oxidative stress, thus improving recovery outcomes and preventing long-term complications such as heart failure (Titus et al., 2023). Additionally, advanced imaging techniques such as pH-sensitive fluorescent proteins could enable real-time monitoring of mitochondrial function, providing insights into disease progression and helping tailor personalized treatment strategies (Godtliebsen et al., 2023). However, challenges remain, particularly in the development of safe and effective drug delivery systems to target mitochondria precisely. Advancements in nanoparticle-based delivery methods will be key to improving precision and safety, while the variability in patient responses calls for personalized treatment strategies. The development of biomarkers to predict patient responses will be essential for optimizing therapeutic outcomes, ensuring that the most effective treatments are delivered.
